# A Good Beginning Makes a Good Market: The Effect of Different Market Opening Structures on Market Quality

**DOI:** 10.1155/2015/307808

**Published:** 2015-08-13

**Authors:** Gernot Hinterleitner, Ulrike Leopold-Wildburger, Roland Mestel, Stefan Palan

**Affiliations:** ^1^Department of Statistics and Operations Research, Karl-Franzens University Graz, Universitatsstraße 15/E3, 8010 Graz, Austria; ^2^Institute of Banking and Finance, Karl-Franzens University Graz, Universitatsstraße 15/F2, 8010 Graz, Austria; ^3^Department of Banking and Insurance, University of Applied Sciences Joanneum, Eggenberger Allee 11, 8020 Graz, Austria; ^4^Department of Banking and Finance, University of Innsbruck, Universitatsstraße 15/4, 6020 Innsbruck, Austria

## Abstract

This paper deals with the market structure at the opening of the trading day and its influence on subsequent trading. We compare a single continuous double auction and two complement markets with different call auction designs as opening mechanisms in a unified experimental framework. The call auctions differ with respect to their levels of transparency. We find that a call auction not only improves market efficiency and liquidity at the beginning of the trading day when compared to the stand-alone continuous double auction, but also causes positive spillover effects on subsequent trading. Concerning the design of the opening call auction, we find no significant differences between the transparent and nontransparent specification with respect to opening prices and liquidity. In the course of subsequent continuous trading, however, market quality is slightly higher after a nontransparent call auction.

## 1. Introduction

The opening of markets is a crucial time in the trading day, because it constitutes the starting point of the price discovery process and the beginning of the incorporation of overnight information into prices. The uncertainty about the fundamental value of the asset is relatively high, leading to lower market quality in terms of mispricing and liquidity compared to intraday trading [1–3]. Therefore, the question of which trading structure is best suited for the opening carries particular relevance.

The study at hand focuses on the effect of opening call auctions on several measures of market quality. The objective is to determine which market structure is ideal for the market opening and whether there exists a spillover effect from an opening call auction on subsequent continuous trading. As will be made clear below, the relevant literature on these issues has come to divergent results. One reason for this fact might be the differences in the applied research methodologies. Our study aims at bridging the gap between the results of empirical and theoretical research by using a stable environment. For three reasons we opt for an experimental approach. (1) When using field data, informational efficiency can only be measured using estimates of the underlying fundamental value. (2) The distribution of information is not known in such empirical studies. (3) Comparing three kinds of market structures in one framework, which is a prerequisite for our research question, is not possible on real stock markets.

In contrast to earlier studies, our examination extends the existing literature in two important respects. First, our study not only investigates the market quality of call auctions at the market opening, but also analyzes the effect of an opening call auction on the subsequent continuous trading phase. We achieve this by comparing a continuous double auction after an opening call auction with a stand-alone continuous double auction. We furthermore analyze two different kinds of call auctions: a transparent design with an open order book and a nontransparent version where no order information is available during the order entry phase. We separately evaluate the effects of these two specifications concerning market quality for both, market opening and subsequent continuous trading.

We find that both versions of the opening call auction significantly increase the informational efficiency of opening prices relative to the stand-alone continuous double auction. Regarding the effect of an opening call auction on the subsequent continuous trading phase, we discover a positive spillover effect in terms of a higher liquidity compared to the single trading venue without an opening call auction.

We also find that the transparent version of the call auction does not lead to a more efficient opening price, nor generate higher liquidity at the opening, than the nontransparent call auction. The continuous double auction after the transparent opening auction furthermore exhibits a partly lower market quality than the continuous trading phase after the nontransparent call auction.

This paper is organized as follows: Section 2 reviews the literature and describes the experimental design and Section 3 presents and discusses the results. The paper concludes with some suggestions for future research on call auction and market opening issues.

## 2. Materials and Methods

### 2.1. Literature Review

Historically, many stock exchanges employed a continuous trading mechanism for their most liquid securities over the entire trading day. Nowadays, however, most western capital markets have switched to using a call auction as their opening mechanism. The most frequently advanced argument in support of this focuses on the ability of call auctions to collect orders and pool liquidity by including different orders from different investors. One of the first studies confirming this argument is that by Madhavan [4], who finds that in the case of high information asymmetry, a call auction structure is best suited in terms of informational efficiency and liquidity. He concludes that a call auction should be used at difficult trading times such as the market opening. Along this line, Economides and Schwartz [5], Domowitz and Madhavan [6], and Pagano and Schwartz [7] point out that in call auctions transaction costs are reduced compared to continuous trading structures because of lower adverse selection costs and because of the absence of a free trading option (A free trading option is created when a trader enters a limit order in the continuous double auction, thus giving other traders the opportunity to buy the stock at the limit price. If stock prices suddenly shift in the wrong direction and the initiator is not able to change the limit price in time, the initiator may realize a loss. This source of trading risk is eliminated in the call auction, since all trades occur at the same time at one market clearing price, cf. Stoll [8]). The advantages of call auctions are also supported by Pagano and Röell [9], Snell and Tonks [10], and Aitken et al. [11] among others.

Ellul et al. [12] document that price discovery at the opening on the London Stock Exchange is more efficient in the call auction than in the dealer market, although the call auction is associated with higher transaction costs, especially in the presence of high informational asymmetries and great order imbalance. Comerton-Forde et al. [13] analyze the introduction of a call auction at the Singapore Stock Exchange and find that informational efficiency is enhanced relative to the mechanism previously in place, which employed a continuous double auction.

Beside the mentioned theoretical and empirical investigations, several studies investigated the question of market opening in an experimental framework. Noussair and Tucker [14] and Palan [15] provide two recent reviews of the literature on experimental market research. The paper which comes closest to our study is Theissen [16]. He compares a call auction, a continuous double auction and a dealer market. He discovers that a call auction does not lead to lower informational efficiency compared to the continuous double auction but generates higher liquidity. The worst performance in terms of market quality is exhibited by the dealer market.

Note that the analyses above do not investigate the influences of different designs of opening call auctions on market efficiency. Madhavan [17] and Pagano and Röell [18] compare a nontransparent call auction, in which market participants have no information about the order book, with a transparent version. Both conclude that the latter should lead to higher market quality due to the greater amount of information revealed during the bidding process. Analyzing the preopening phase of transparent call auctions, Biais et al. [19] and Davies [20] show that market quality is higher in the later stage of the preopening phase compared to the beginning at the Paris Bourse and the Toronto Stock Exchange, respectively. In contrast to these results, Madhavan et al. [21] report that higher transparency at the Toronto Stock Exchange proved detrimental to market quality. From this Eom et al. [22] conclude that the evidence on the effect of pretrade transparency on market quality is mixed.

Beside the impacts of different market opening designs on market efficiency and liquidity at the beginning of the trading day, some investigations deal with the question whether a specific market opening design may also influence market quality in the subsequent continuous trading phase “spillover effect”. Since different stock exchanges have been using different specifications of call auctions, questions concerning, for example, the transparency during the preopening phase or the shape of priority rules, have attracted increased attention in the literature. Chang et al. [23] report positive spillover effects after the introduction of an opening call auction at the Singapore Stock Exchange. Pagano et al. [24] show that these results extend to NASDAQ. Comerton-Forde et al. [13] on the other hand document that market quality declined after the introduction of an opening auction at the Hong Kong Stock Exchange. This decline is accompanied by increasing spreads, which in turn lead to reduced liquidity. The authors attribute this problem to an unsuitable design of the opening mechanism which renders it unattractive for traders.

Gerace et al. [25] analyze the spillover effect of a more transparent opening call auction on continuous trading at the Shanghai Stock Exchange (SHX). The authors report that the volatility and spreads during intraday trading were reduced after the introduction of indicative prices. This general result receives further support from Hoffmann and van Bommel [26], who compare stocks listed at the more transparent Euronext Paris with ones listed at the slightly less transparent German Stock Exchange. In their analysis, informational efficiency and liquidity in continuous trading is higher at the Euronext Paris.

We thus conclude our review of the relevant literature by identifying the gap we intend to fill. No studies have hitherto analyzed a call auction and a subsequent continuous double auction in a unified framework, investigating both the direct and the spillover effects, and complementing this investigation by analyzing the differential impact of transparency. This is what we will address.

### 2.2. Experimental Design

In total, we conducted 21 sessions from October 2009 to November 2010, using three treatments with seven sessions each. The experiment was programmed in z-Tree [27]. Each session involved 12 participants from the student body of the Faculty of Social and Economic Sciences at the Karl-Franzens-University Graz, recruited using the ORSEE system [28]. Each participant took part only once in this experimental setting.

#### 2.2.1. Description of Treatments

The experimental design is based on the work of Theissen [16], who compares three different stand-alone trading structures. In contrast to this investigation, we have a five-minute stand-alone continuous double auction (CDA) and two different types of complement markets, both starting with a two-minute call auction. After the opening price has been determined, a five-minute continuous double auction begins also in these treatments.

The two complement markets differ with respect to the transparency level of the opening call auction. In the first complement market (CM) a completely nontransparent call auction is used, meaning that no information about the order flow or the indicative prices is available to the participants. The second complement market (CMT) employs a fully transparent call auction; that is, market participants can see the whole order book and the indicative prices during the entire duration of the preopening phase. (Note that these two kinds of call auctions occupy the two extremes in the continuum of possible transparency levels. Real stock markets are in general located somewhere in between these transparency levels, although there are exceptions like, e.g., the Paris Bourse, which employs a fully transparent call auction (cf. [29]).) Furthermore, the indicative prices are steadily being updated during the order entry phase. During the preopening phase, participants are allowed to enter and change (e.g., cancel) their orders. To enter an order the trader has to enter a price and a volume on the selected market side.

#### 2.2.2. Market Mechanisms

In the call auction, the price which leads to the highest trading volume is used for both indicative prices and the opening price. In the case of two or more prices with the same trading volume, the middle of the price range leading to the maximum trading volume is used. Generally, buy (sell) orders with prices higher (lower) than the opening price are being executed. If one market side exceeds the other in terms of order volume at the opening price, a rationing mechanism is applied. In contrast to other experiments (e.g., [16, 30]), this rationing mechanism uses a price-time-priority rule like in Friedman [31]. If two or more traders on one market side have the same price limit, the order entered first is processed first. At the opening all participants receive information about the opening price, the trading volume, and the order surplus.

The continuous double auction has the same design in all treatments. The best bid and ask prices are visible to all participants. A new bid (ask) is permissible if its price is higher (lower) than the current bid (ask) price. If a new bid or ask order is entered and accepted, all orders entered earlier are deleted. A transaction is executed if a trader accepts the current bid or ask price. The order entry is again organized such that participants have to enter price and volume on the selected market side. If a trader wants to accept a current bid (ask) she only has to enter an order volume of between 1 and the volume of the bid (ask) in the market.

#### 2.2.3. Session Structure and Payoffs

Each session of the stand-alone continuous double auction (complement market) lasts approximately 120 min (160 min). First, the participants get instructions explaining the trading rules, the structure of the experiment, the payoff, and details about the traded asset and the information structure. Next they complete a self-test in which they have to answer questions concerning the instructions. If a participant gives an incorrect answer, the experimenter is informed and helps the participant in order to achieve a level playing field in terms of subject understanding.

In each experimental session there are 8 trading periods, the first two of which serve as training periods in which participants are able to get acquainted with the trading interface. These training periods do not count towards subjects' earnings, which are based only on subjects' performance in the following six trading periods. (After these 8 trading periods we conducted a second experimental treatment. At the beginning of the session, subjects were made aware that there would be a second part but did not learn any details about it.) This is communicated to the students at the beginning of the experiment and is additionally documented in the written instructions they receive. Each period is structured as follows: the individual price signals are displayed on subjects' screens and subjects have 20 seconds to contemplate them before trading starts. In the complement markets, subjects then have 120 seconds in which to submit call market bids and asks. After the 20 seconds have elapsed in the CDA, the continuous trading phase starts. After the 120 seconds have elapsed in the complement markets, the opening price is determined from the submitted bids and asks and subjects are informed about the price and about how many shares they have traded. The information screen is displayed for 10 seconds, after which the continuous trading phase starts. After five minutes this phase ends and subjects' endowment of experimental currency and cash is reset to their initial values, after which trading restarts for the next period. (At the end of a trading period, subjects do not receive any additional information. In particular, they are not informed about their trading profits or the fundamental value.)

As in Theissen [16], each participant's initial portfolio consists of 50 000 currency units and 100 shares and is reinitialized at the beginning of every period. Subjects can sell short up to 100 shares and can overdraw their currency account by up to a maximum of 50 000 currency units. No interest and dividends are being paid in the experiment. Therefore, the profit of a participant results exclusively from the prices and quantities of her trades and from the fundamental value of her shares at the end of the experiment. The minimum tick size is one experimental currency unit.

The payment for a participant consists of a fixed show-up fee plus an amount depending on the sum of her six period-end portfolio values. The payment for the trading success is exponentially structured depending on the trader's portfolio value rank within the session. The performance-dependent payout is shown in Table 1. The payoff structure thus conveys a clear incentive to trade.

A second reason for this type of payout function is that it allows for relatively large marginal incentives, while ruling out the possibility of bankruptcy. When allowing for short selling and margin buying, a payoff function linear in subjects' terminal wealth or profit admits the possibility of negative final wealth. In order to circumvent this problem, one strategy would be to add a fixed lump-sum payment to the outcome of the experiment and use an exchange rate from experimental into real currency which ensures that bankruptcy is very unlikely. However, in this case the marginal incentives from performing well in the experiment are relatively small, since the difference in payouts to good and bad performers is—by necessity—small. Our payoff function allows for large marginal incentives (i.e., great differences between the best and worst performers) without the risk of bankruptcy. Furthermore we argue that convex incentives and incentives determined by a trader's rank relative to others are not uncommon in markets outside the laboratory. Brown et al. [32], for example, argue that the market for mutual funds can be considered a tournament, since past performance (relative to others) drives new investment inflow and because fund managers' rewards are usually tied to the assets under management. (“Specifically, we suggest that viewing the mutual fund market as a tournament in which all funds having comparable investment objectives compete with one another provides a useful framework for a better understanding of the portfolio management decision-making process. Similar to the payoffs for golf and tennis competitions, the amount of remuneration that a fund receives for ‘winning' this tournament depends upon its performance relative to the other participants” [32].) Similarly, Basak et al. [33] analyze this link between fund flows and performance, noting that it induces convexities in managers' objectives. A number of recent studies are thus motivated by the importance of tournament and convex incentives in real-world financial markets and analyze their impact on trader behavior and market outcomes (further examples include [34–37]).

In addition to the variable payoff, every participant receives a 10 € show-up fee in the complement market and an 8 € show-up fee in the continuous double auction setting. The differences in payoffs stem from the fact that the complement markets have a longer time duration of about 30 minutes compared to the setting with the continuous double auction alone. On average, the participants of the different treatments receive identical expected payoffs of 8.50 € per hour. After the experimental periods have concluded, participants have to fill in a questionnaire dealing with the experiment in general and the behavior of the individual in particular.

Each period's fundamental value *V*_*t*_ is independently drawn from a discrete uniform distribution over all integer values in the interval [0.8 · *V*_*t*−1_, 1.2 · *V*_*t*−1_], (Due to a programming error, in 1.85% of all cases (i.e., in the second period each of one stand-alone CDA and one nontransparent complement market) the fundamental value changed by 24.1% from one period to the next. Note, however, that subjects were only informed about the last period's fundamental value.) The first asset value of the six trading periods is randomly chosen from the interval of 400 to 600 currency units. (The asset values in the two training periods were 86 and 90. They were chosen far from the range of values employed later for the six periods which counted towards payoffs in order to eliminate the possibility of anchoring.) The fundamental value is not communicated or displayed to the participants at any time during or after a trading period, except for the very end of trading (i.e., after period 8). Instead, each participant receives an individual, imperfect price signal for the fundamental value of the asset before a trading period starts. In line with the calculation of the fundamental value, the price signals are uniformly distributed over all integer values between 10% below and above the fundamental value. (During the pretests of the final experimental setting we also conducted some series with a higher range of price signals and therefore a higher information asymmetry. The results do not indicate that this change in the price signal distribution is associated with a change in market quality. Due to a programming error, in 3.49% of all cases the signals deviated by more than 10% (maximum: 12.01%) from the fundamental value. Since subjects did not learn the fundamental value in periods 1 through 5, they could have become aware of the problem in 0.47% of all cases (i.e., when a signal's deviation was too large in the last period), and only at the end of the experiment after trading had concluded. No subject gave an indication of having become aware of one of these cases.) Therefore, the price signal is of equal ex-ante quality for all traders, whereas the ex-post signal quality differs in terms of precision between the traders. We do however ensure that, within each period, the average of the randomly calculated price signals equals the fundamental value of the asset. The variation in price signals—computed as the average absolute deviation of each of the 12 price signals from the fundamental value of the period—ranged between 3% and 6.5%. The average absolute variation of price signals over all periods was 4.8%. All the facts concerning the information structure within the experimental market were common knowledge.

## 3. Results and Discussion

The results we present are based only on the six experimental trading periods. We structure the presentation into results regarding the market opening, regarding the preopening phase and regarding possible spillover effects.

### 3.1. Market Opening

We start by analyzing the informational efficiency of the opening prices of all treatments and obtain the following result.

*Result 1*. An opening call auction prior to continuous trading leads to a smaller opening price deviation from fundamental value and lower spreads than a continuous double auction alone. This result holds for both transparent and nontransparent call auction forms.

*Support for Result 1*. The mean relative error (MRE) for the opening prices *P*_*o*,*t*_ in a series  measures the average absolute difference between these prices and the fundamental value *V*_*t*_, divided by the fundamental value (cf. [16]). We first define the relative error (RE) as follows:

(1) $$\begin{array}{c} {RE}_{o,t}=\frac{\left|P_{o,t}-V_{t}\right|}{V_{t}}. \end{array}$$

The MRE for the opening prices is then defined as the session average of the individual periods' RE_*o*,*t*_ values as follows:

(2) $$\begin{array}{c} {MRE}_{o}=\frac{1}{6}\cdot \overset{8}{\underset{t=3}{\sum }}{RE}_{o,t}. \end{array}$$

Here, *t* is the trading period, running from 3 to 8 to exclude the training periods 1 and 2. Lower MRE_*o*_ values indicate higher informational efficiency of the opening price. Using the MRE as a measure of informational efficiency of a market, we find that the call auction leads to a higher level of efficiency than the continuous double auction in terms of the opening price. We obtain MRE_*o*_ values of 2.61% (1.92%) [3.93%] in the transparent call auction (nontransparent call auction) [stand-alone CDA]. To investigate the robustness of these differences, we conduct a number of statistical tests. Since the assumption of a normal distribution is rejected for various datasets in our analysis, we report only nonparametric tests. It is worth mentioning that several hypotheses were also tested by use of parametric methods, yielding qualitatively equivalent results as the reported nonparametric tests.

The differences between the MRE_*o*_ values of the three treatments are investigated by a Kruskal-Wallis-ANOVA which shows that they differ significantly (*P* value: 0.035). In addition we execute Wilcoxon rank sum tests comparing the MRE_*o*_ values of the stand-alone CDA to those from the call auctions. The comparison between the opening prices of the stand-alone CDA and the nontransparent call auction shows that the latter generates significantly more efficient opening prices (*P* value: 0.013). Comparing the prices from the transparent call auction to either the opening prices of the stand-alone CDA or the nontransparent call auction prices yields no significant differences (*P* values: 0.142 and 0.225, resp.).

Finally, we compare the MRE_*o*_ values of the call auctions to the MRE 120 seconds after the start of the stand-alone CDA. This comparison yields evidence on the competitive performance of these different opening mechanisms, controlling for the time cost (i.e., the call auctions and the stand-alone CDA in this comparison have both been given 120 seconds to aggregate prices). The average MRE 120 seconds into the stand-alone CDA is 3.56%, which is significantly (not significantly) different from the MRE_*o*_ yielded by the nontransparent (transparent) call auction (Wilcoxon rank sum test* P* values: 0.048 and 0.225).

However, these tests suffer from a lack of power because they must compare session averages of the RE_*o*,*t*_ values for the six periods in each session (i.e., MRE_*o*_), since the results from individual periods within a session are not independent of each other. To remedy this shortcoming and make better use of our data while still accounting for the possible dependence between consecutive periods within a session, we regress RE_*o*,*t*_ on a constant, on dummies for the two treatments using either transparent or nontransparent call auctions, and on dummies for the period within a session, for the sessions themselves, and for the interactions of periods and sessions, adjusting for clusters in the standard errors by session:

(3) REo,t=α+βCM·CM+βCMT·CMT+βtT·t+βsT·s+βt×sT·t×s+εt.

In this regression, CM (CMT) is a dummy variable equal to 1 in the nontransparent (transparent) call market treatment and zero otherwise. The expressions **β**_*t*_^*T*^, **β**_*s*_^*T*^, and **β**_*t*×*s*_^*T*^ are transposed coefficient vectors, **t** is a vector of dummies for periods 4 through 8, **s** is a vector of dummies for sessions 2 through 7 and **t** × **s** is the interaction between periods and sessions. The results are shown in regression model (1) in Table 2. We find significant treatment effects for both the transparent and the nontransparent call auctions. We then repeat the analysis, again using RE_*o*,*t*_ for the opening prices in the treatments with call auctions, but using the relative error after 120 seconds in the CDA. The results, reported in model (2) in Table 2, are similar, albeit with slightly smaller (absolute) coefficients. We interpret this as evidence that the existence of a preceding call auction of either transparency level leads to a more efficient opening price than in the case of a stand-alone CDA.

The homogeneity of the call auctions' efficiency is also supported when analyzing the liquidity of the two types of call auctions and the stand-alone CDA. As in most empirical (e.g., [26, 38]) and experimental studies (e.g., [16, 31]), we use the bid-ask spread as a measure of market liquidity. We define the spread in the CDA to be the difference between the lowest ask and the highest bid in the order book at the time when a transaction takes place. (Remember that our order book has a depth of 1, since subjects can only enter orders which improve the quoted spread (i.e., are higher than the previous highest bid and lower than the previous lowest ask) and that previous bids (asks) are deleted upon entry of a new best bid (ask). For this reason, in 17 out of 42 cases no spread exists at the time of the opening transaction in the stand-alone continuous double auction.) In a call auction, however, no direct bid-ask spread exists. We therefore follow Friedman [31] and Theissen [16] in calculating the relative inside spread as

(4) $$\begin{array}{c} {RIS}_{t}=\frac{P_{o,t}^{A,l}-P_{o,t}^{B,h}}{V_{t}}, \end{array}$$

where *P*_*o*,*t*_^*B*,*h*^ (*P*_*o*,*t*_^*A*,*l*^) is the highest bid (lowest ask) price of a buy (sell) order which is not executed in the call auction. The difference between these two prices is an indirect spread, because an increase (reduction) of *P*_*o*,*t*_^*B*,*h*^ (*P*_*o*,*t*_^*A*,*l*^) to the level of *P*_*o*,*t*_^*A*,*l*^ (*P*_*o*,*t*_^*B*,*h*^) would lead to the execution of the order. An additional order can only be executed at a price between *P*_*o*,*t*_^*B*,*h*^ and *P*_*o*,*t*_^*A*,*l*^, implying that RIS_*t*_ represents the price impact of an additional order. We find that the average spreads of the opening call auctions are 2.28% for the nontransparent call auction and 2.14% for the transparent call auction (medians: 2.62% and 3.27%, resp.), a difference which is not significant in a Wilcoxon rank sum test (*P* value: 0.848). (The same result obtains when analyzing the trading volume at the opening (*P* value: 0.678). Note however that trading volume is a relatively poor measure of liquidity because no dimensions of liquidity are impounded (e.g., [39]).) The average spread in the CDA, conversely, is 4.52% (median: 4.25%). Applying again the regression methodology outlined in (3) but using as the dependent variable the relative inside spread in the case of the call auction treatments and the direct spread in the stand-alone CDA, we find and report in model (3) of Table 2 highly significant treatment coefficients.

Summing up our results from the market opening, we find support for earlier findings by Madhavan [4], Economides and Schwartz [5], and Comerton-Forde et al. [40]. They show that an opening call auction seems to be best suited for difficult trading situations like the market opening, when trader uncertainty about the fundamental value is high. The attributes of the call auction with regard to collecting orders, generating liquidity, and incorporating heterogeneous orders and traders with different expectations and information therefore tend to lead to a more efficient opening price in such settings. Three findings strengthen the conclusion that this effect is caused by the mechanism of trading (i.e., call versus continuous double auction). First, the effect is robust to analyzing prices after the same amount of time has elapsed in the three treatments. Second, the effect is present in both transparency levels of the call auction, even though the transparent call auction and the continuous double auction provide traders with a comparable level of information which is considerably greater than that in the nontransparent call auction. Third, the results hold both for price efficiency and for the spread.

As a second high-level observation we note that we detect no significant differences between the transparent and the nontransparent call markets regarding any of the measures investigated so far. If there is a difference, our results suggest that it would favor the nontransparent version, which has higher (absolute) coefficients and lower* P* values in most analyses. This result is surprising. In contrast to most of the literature, which finds efficiency increasing in transparency (e.g., [22, 41–43]), relatively few studies report similar results regarding the issue of transparency in call auctions (e.g., [21, 44]).

### 3.2. Preopening Phase

In order to analyze our results at market opening in more detail, we investigate the development of prices and the order flow during the preopening phase. Such an analysis has the potential to shed more light on the question of how the different specifications of call auction mechanisms perform. We obtain the following result for the preopening phase.

*Result 2*. In both forms of the opening call auction (transparent and nontransparent), price discovery is completed within the first minute of order submission.

**Figure 1:**
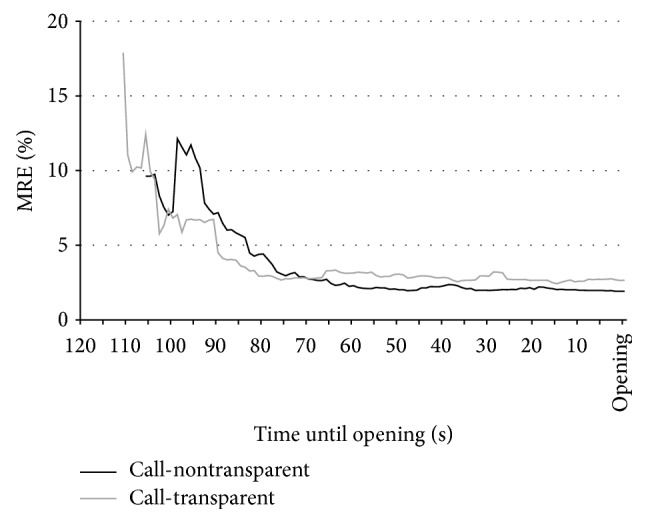
Average MRE of indicative prices in the call auctions. This figure displays the development of the average mean relative error (MRE) in the transparent and nontransparent call auctions over time.

*Support for Result 2*. Our experimental framework enables us to compare a transparent auction mechanism with a nontransparent version with respect to the preopening behavior. Although the participants do not receive any information concerning the order flow and the indicative prices in the latter, we are nonetheless able to compute the indicative prices in this auction mechanism. The amount and variety of orders and market depth in both auction forms increase with increasing time elapsed in the preopening phase. As Figure 1 illustrates, both call auction forms have completed the price discovery process by the end of the first minute of order submissions. Furthermore, they achieve a similar level of informational efficiency, measured by the MRE of the indicative prices.

To further investigate this topic, we compute the MRE for the first indicative prices and for the indicative prices in the middle of the preopening phase (after 60 seconds). (In addition to computing the MREs of the first indicative prices we analyze the time at which the first indicative price becomes available in the call auctions. In the transparent (nontransparent) call auction the first indicative price is arrived at on average after 23.5 (24.2) seconds. A two-sample* t*-test on a period-by-period basis shows that this difference is not significant (*P* value: 0.596) and linear regression analysis shows that the duration until the appearance of the first indicative price does not influence the MREs of the first indicative prices, nor the mid MREs or the opening MREs.) Afterwards we compare these MRE values to the ones at market opening. Again, these three MRE values are calculated as averages over all trading periods, which leads to 7 observations per treatment and type (first indicative price, indicative price in the middle of the preopening phase, and opening price).

Using a Kruskal-Wallis-ANOVA of the MRE values per experimental session we find significant differences between the distributions of the three price types for both the transparent (*P* value 0.020) and the nontransparent call auctions (*P* value: 0.001). The results of Wilcoxon rank sum tests show that the MREs of the first indicative prices are significantly greater than the MREs of the indicative prices after 60 seconds in the transparent (*P* value: 0.048) as well as the nontransparent call auctions (*P *value: 0.002). The same is true for the comparison of the MREs of the first indicative prices to the opening price MREs (*P* values: 0.009 and 0.003), respectively. The main result of higher price efficiency at the end of the preopening phase compared to the beginning is in line with [19], who find a similar result for a transparent call auction preopening mechanism at the Paris Bourse.

**Figure 2:**
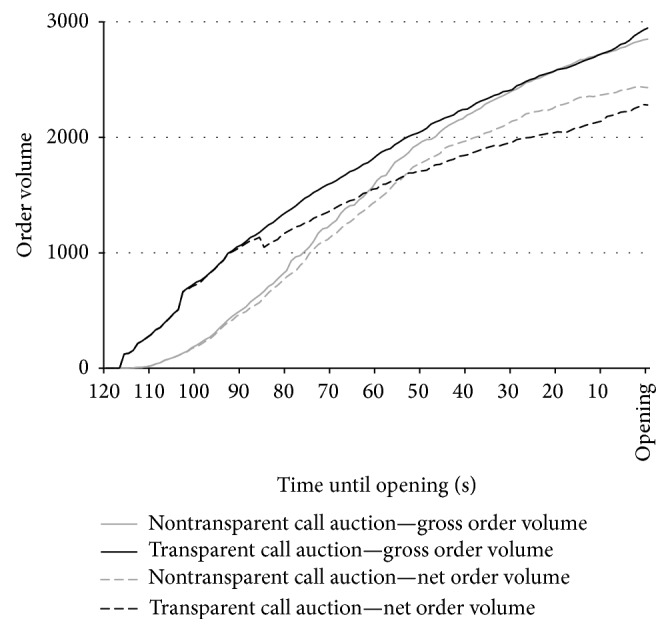
Gross and net average order volume during the preopening phase. This figure displays the development of the average order volume in the transparent and nontransparent call auctions gross and net of cancellations over time.

Figure 1 illustrates graphically that the price discovery process is less pronounced (or even nonexistent) from the middle of the preopening phase to the opening. More technically we find that the MRE of the opening prices is different from that of the indicative prices in the middle of the preopening phase for neither the transparent nor the nontransparent call auctions in Wilcoxon rank sum tests (*P* values: 0.406 and 0.225). This conflicts with the result of Biais et al. [19], who report efficient price discovery especially in the later part of the pre-opening phase. One reason for this discrepancy may be that in our experimental study, a price-time priority rule is implemented, possibly leading to a higher order volume already at the beginning of the preopening phase (see Figure 2). The Paris Bourse however did not have such a rule during the sample period. This may have led to the higher trading activity in the later part of the preopening phase and the more efficient price discovery process documented by Biais et al. [19].

In accordance with the results from the opening prices, we observe no significant differences between the first or mid-period indicative prices between the two treatments of the call auction (*P* values: 0.565 and 0.180 in Wilcoxon rank sum tests), suggesting again that a transparent market, although providing more information about the market situation, does not lead to higher market quality compared to the nontransparent auction. (As Figure 1 indicates, the marginally significant difference between the mid-period MREs suggests that, on the contrary, the nontransparent market is more efficient (average MRE: 2.29%) than the transparent market (average MRE: 3.09%).) A closer look at the preopening order flow allows us to investigate if and under which circumstances the market quality in the transparent call auction differs from that in the nontransparent setting. Figure 2 displays an overview of the order flow during the preopening phases of both call auctions. It shows the gross and net average order volumes separately, with net order volume in this context defined as the gross order volume minus the volume of cancelled orders.

Figure 2 highlights slight treatment differences. At the beginning of the preopening phase, the (cumulative) gross order volume is greater in the transparent auction, but the nontransparent auction order volume catches up over time. The net order volumes under the two treatments exhibit a similar development as the gross volumes early in the preopening phase (this is to be expected, since order cancellations accrue over time and must by definition lag order entries), but then intersect in the middle of the preopening phase. Finally, cumulative net order volume ends up being significantly higher in the nontransparent auction at the time of the opening. This shows that, during the second half of the preopening phase, more orders are being cancelled in the transparent than in the nontransparent call auction. The higher incidence of order cancellations in the transparent auction may hint at strategic behavior in the sense that subjects submitting orders which are later cancelled may not really intend to trade at the submitted prices but could be trying to move the market through the revelation of what others may believe to be information (cf. [31]). In line with Davies [20], we compute the fraction of cancelled orders which would have been executed at the indicative price. For the second half of the preopening phase we find this to be 3.27% (5.94%) in the nontransparent (transparent) auction. (For the first half of the preopening phase the average fraction of cancelled quotes is 2.39% (3.80%) for the nontransparent (transparent) call auction. This difference is not statistically significant in a Wilcoxon rank sum test (both* P* values: 0.220).) A Wilcoxon rank sum test confirms that this difference is statistically significant (*P* value: 0.022). However, a second interpretation of the greater number of order cancellations in the transparent treatment would be that there are greater learning effects, which cause subjects to rethink and cancel their orders. Since our data are insufficient for further pursuing this issue, we leave it for future research.

### 3.3. Spillover Effects

In the majority of real stock markets, trading after the opening call auction continues in the form of a continuous double auction. This raises the question whether an opening call auction not only leads to a more efficient opening price, but also influences subsequent continuous trading in a positive manner in terms of market quality. Our experimental research design enables us to analyze these spillover effects of different call auction formats in a common framework by comparing measures of market quality from a double auction market preceded by a call market with data from a stand-alone double auction market.

We start our investigation by analyzing the price efficiency during the continuous double auction. In order to do so, we employ the following adapted RE and MRE measures:

(5) $$\begin{array}{c} {RE}_{t}^{CDA}=\overset{n}{\underset{i=1}{\sum }}\left(\frac{\left|P_{i,t}-V_{t}\right|}{V_{t}}\cdot \frac{S_{i,t}}{\sum_{i=1}^{n}S_{i,t}}\right), \end{array}$$

(6) $$\begin{array}{c} {MRE}^{CDA}=\frac{1}{6}\cdot \overset{8}{\underset{t=3}{\sum }}{RE}_{t}^{CDA}. \end{array}$$

In (5) we first calculate the period average of the relative error (RE_*t*_^CDA^) of each transaction in the continuous double auction in period *t* by taking the relative absolute differences between the transaction prices and the fundamental value and—in contrast to the call auction—weighting them by the proportion of the period's total trading volume represented by the transaction in question (cf. [16]). The weighting factor serves to limit the influence transactions small in volume but large in price deviation have on the overall MRE of a continuous trading period (MRE_*t*_^CDA^). In a second step we derive the MRE^CDA^ as the average of the sum of the RE_*t*_^CDA^ values of all transactions in a period, over all periods for a series, as presented in (6).

We list the mean, median, and standard deviation of each treatment's MRE^CDA^ values in Panel A of Table 3. The notation we use is as follows: “CDA-CM” stands for the continuous double auction after the nontransparent call auction; “CDA-CMT” is the continuous double auction after the transparent call auction; and “CDA alone” is the stand-alone continuous double auction without an opening auction.

We use Wilcoxon rank sum tests to compare the stand-alone continuous double auction outcomes to data from the markets preceded by either of the two types of call auctions. The results are reported in Panel B of Table 3. Despite our small sample size of only seven observations each of the results indicates that prices in the continuous double auction are more efficient subsequent to a nontransparent opening call auction than without such an opening institution. The difference between the stand-alone CDA and the CDA-CMT is not significant. When using only the MRE calculated over the last transaction price in each session of the CDA, we find no significant results.

We obtain a stronger result when we again estimate the regression specification from (3), using RE_*t*_^CDA^ as the dependent variable. The treatment dummy coefficients for the CDA following both the nontransparent and the transparent call markets, reported in model (4) in Table 4, are negative and significant. This would support the assumption that opening call auctions reduce uncertainty about the fundamental value, thus indicating that a good start of trading goes hand in hand with an overall increase in market quality.

**Figure 3:**
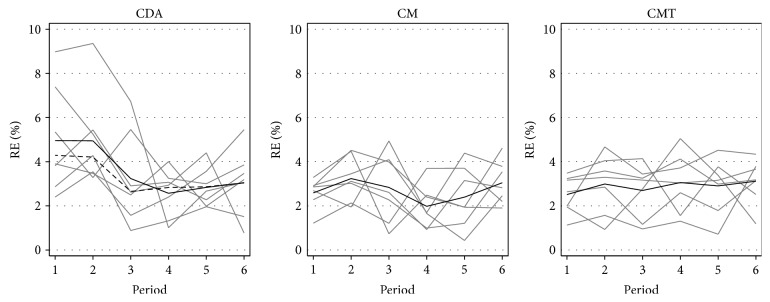
Average RE^CDA^ in the CDA phase, by treatment. This figure displays the development of the average relative error in each session's CDA phase (RE^CDA^), in each of the three treatments (thin grey lines), the mean over all sessions (solid black line), and the mean over all sessions without the first session in the case of the stand-alone CDA (dashed black line, leftmost panel).

While these findings appear promising at first sight, we believe that they actually constitute a result of learning by the market participants, which leads the spillover effects to disappear in repeated markets. To substantiate this claim, we plot the average value of RE^CDA^ by period in order to give an overview of the heterogeneity and average development over time. We do so in Figure 3, which displays the development of RE^CDA^ in each session, in each of the three treatments, depicted in thin grey lines. The solid black line is the mean over all sessions. Since the first CDA session exhibits relatively high mispricing in the first three periods, we also calculate the mean without this session. The dashed black line in the left panel displays the mean without this possible outlier, documenting that prices remain relatively high early in the CDA markets.

The overall patterns in Figure 3 clearly document relatively large heterogeneity between sessions, but relatively similar convergence in later periods. While the CM and CMT treatments seem to follow similarly flat trajectories, the panel for the stand-alone CDA suggests the existence of a learning effect from the earlier to the later periods. We therefore test for differences in the RE^CDA^ values between treatments and provide the results in Table 5. (We also repeat the regression analysis excluding periods 1 and 2, finding that the significant treatment effect likewise disappears.)

The results indicate that mispricing is indeed significantly higher in the early periods of the CDA treatment (and that this effect is relatively robust to the removal of one possible outlier, despite the sample size of only 7 sessions each). Nonetheless, our findings also show that the positive spillover effect from the opening call auctions disappears after the second period of the market. The reason is not that prices in the CDA following a call auction become less efficient but rather that prices in the stand-alone CDA become more efficient. Given that we run two training periods, we interpret this as evidence that our market participants require substantial experience to largely eliminate mispricing in this setting. When they gain this experience, however, the difference between our treatments disappears. In other words, our spillover effect findings seem to be driven by an interaction with subject experience, with a call auction ameliorating the pricing errors in markets with inexperienced subjects.

We find no such outliers or clear time trends when analyzing market liquidity. In line with the outcomes for MRE^CDA^, we perform a comparison of the spreads (In contrast to the call auction, spreads are directly observable in the continuous double auction by computing the difference between ask and bid prices when a transaction occurs. They are particularly relevant in this setting, since transactions tend to take place when spreads are lower and because traders are affected by the spreads only when a transaction occurs (cf. [16]). Note that no spread can be derived when somebody accepts the standing bid (ask) at a time when there is no ask (bid) in the market.) between the continuous double auction alone and the continuous double auctions of the complement markets. Descriptive statistics as well as results of the Wilcoxon rank sum tests are shown in Table 6 (Panels A and B, resp.).

Table 6 documents that the spreads in the continuous double auction after a nontransparent opening call auction are significantly lower than in the stand-alone continuous double auction. We again estimate the regression specification from (3), using the average spread as the dependent variable. The results are reported in model (6) in Table 4. Our results show the treatment dummy coefficient for the CDA following the nontransparent call market to be negative and significant, while the coefficient for the CDA after a transparent call market is not significant. This supports the assumption that a nontransparent opening call auction reduces uncertainty about the fundamental value, leading to higher liquidity in continuous trading after such a call auction.

Note that some traders in the continuous double auction after a transparent opening call auction enter order prices which are particularly far away from other traders' bids or asks and from the fundamental value. Although a majority of these orders is not accepted, they may lead to high reported spreads when an order of the other market side is accepted (since the spread is defined as the difference between the standing bid and ask at the time of a transaction).

This phenomenon becomes apparent when analyzing the intraday volatility in the continuous double auction. The intraday price standard deviation was computed for every period and session. The average is 2.26% (2.47%) [3.12%] in the CDA-CM (CDA-CMT) [stand-alone CDA]. The hypothesis of the existence of positive spillover effects on price volatility is not supported by the Wilcoxon rank sum test results for either the nontransparent opening call auction (*P* value: 0.110) or the transparent version (*P* value: 0.277). We also fail to detect a significant difference between the continuous auctions following the two call auction types (*P* value: 0.848). However, employing our more powerful regression methodology, we can isolate negative and significant treatment effects from the nontransparent (coefficient: −0.0086,* P* value: 0.002) and the transparent call auctions (coefficient: 0.0065,* P* value: 0.019). Interestingly, we find small but significant downward trends when regressing volatility on time in CDA-CM and CDA, but not in CDA-CMT (coefficients: −0.0016, −0.0054, and −0.0010; *P* values: 0.020, 0.002, and 0.281, resp.).

Finally, we again find no significant differences in total trading volume between the three continuous double auctions using the Wilcoxon rank sum test (*P* values: 0.277, 0.142, and 0.565 when comparing the stand-alone CDA and the CDA-CM, when comparing the stand-alone CDA and the CDA-CMT, and when comparing the CDA-CM and the CDA-CMT, resp.). Once more, using the regression methodology, we obtain positive and significant treatment effects from the nontransparent (coefficient: 233.43,* P* value: 0.007) and the transparent call auctions (coefficient: 245.55,* P* value: 0.004).

To further investigate the question of spillover effects, we perform a linear regression analyzing the impact of market efficiency at the market opening after a call auction on subsequent efficiency using the following functional form, with the symbols defined as in (3), and again adjusting for clusters in the standard errors by session:

(7) REtCDA,θ=α+βRE·REo,tθ+βCM·CM+βCMT·CMT+βtT·t+βsT·s+βt×sT·t×s+εt.

In this regression, *θ* ∈ {CM, CMT} indicates the nontransparent and transparent call market treatment, respectively. This regression investigates the association between the opening price efficiencies in the call auctions and the average price efficiencies over the course of the following continuous double auctions and whether there is a treatment difference in the spillover effect between the transparent and the nontransparent versions of the opening call auction. As shown in model (5) in Table 4, we find *β*_RE_ to be highly significant, while the coefficient dummies of the nontransparent (transparent) call market treatments turn out to carry the correct sign but are not significantly different from zero. (This result is robust to excluding the first two periods.) In other words, lower (higher) efficiencies at the opening of the transparent auction market are associated with lower (higher) efficiency in the subsequent continuous double auction, and this effect subsumes any treatment effects that may be present. When we extend this line of analysis to spreads, we find a significant treatment difference but no spillover effect. (When regressing the spreads in the continuous double auction on the spreads in the preceding call market in a specification analogous to (7), we find the results reported for model (7) in Table 4. Regressing the spread in the continuous double auction on the opening MRE from the call auctions yields the findings listed in model (8). As is the case for model (5), RE_*o*,*t*_ also has significant explanatory power for the CDA spread results, while the spread in the call auction preceding the CDA does not. The dummy for the nontransparent complement market treatment is highly significantly different from zero in both models.)

In contrast to some of the empirical literature (e.g., [25, 26]) our experimental results provide no support for the conjecture that a higher level of transparency in the call auction has a positive influence on the market quality in the subsequent CDA. On the contrary, they seem to be more in line with the arguments of Domowitz and Madhavan [6], who show that excessive transparency can have a negative influence on market quality. It is possible that observing the order flow and the price discovery process during the preopening phase increases the feeling of uncertainty in the market, which spills over into subsequent continuous trading. Alternatively it is possible that some participants are overwhelmed by the amount of information revealed in the preopening phase, which would be in line with the concept of “information overload” (cf. [45]; see also [46] for complexity in financial markets and information overload). Finally, we acknowledge the possibility that the lack of a better performance of the transparent call auction is due to the greater room for price manipulation therein, as documented by Arifovic and Ledyard [30]. Unfortunately, our data do not allow us to distinguish between these rival hypotheses.

## 4. Conclusions

The paper deals with the market structure at the opening and its influence on subsequent trading. In contrast to previous papers, we compare a single continuous double auction and two complement markets with different call auction designs as opening mechanisms in a unified framework. This is also the first experimental examination of complement markets using different designs of an opening call auction. The results of our experimental approach can be summarized as follows.

1. A call auction is well-suited as a market opening institution, generating a higher market quality at this time of market stress than opening directly with continuous trading.
2. Opening call auctions cause a positive spillover effect on subsequent continuous trading in terms of spreads, volatility, and trading volume, although not necessarily on mispricing.
3. Higher transparency during the preopening phase leads to higher market quality neither at the opening nor in subsequent trading. While there are no significant differences in terms of market quality at the opening, spreads in the continuous market are significantly higher than in the CDA-CM setting with a nontransparent opening call auction.

We consider this as evidence supporting the decision of most stock exchanges to institute an opening call auction. As predicted by the theoretical literature (e.g., [4, 5]), such an institution seems a suitable instrument at this crucial time of the trading day. Furthermore, the opening call auction has a positive spillover effect on subsequent continuous trading, thereby supporting most of the empirical literature (e.g., [23, 24, 47]). This result also means that an opening call auction creates higher price efficiency and liquidity for the whole market and should therefore lead to a more attractive stock market for issuers and investors, which in turn implies an overall welfare gain for the economy (e.g., [48]). However, as Comerton-Forde et al. [13] pointed out in their examination of the Hong Kong Stock Exchange, the design of call auction markets is a complex issue. It encompasses the choice of a level of transparency during the preopening phase as well as price determination and priority rules. As the present experimental examination shows, the design of the call auction can influence the behavior of traders, with attendant implications for market quality.

In periods with inefficient prices during the first seconds of the preopening phase, traders seem to try to avoid prices getting more efficient by placing orders that support the current inefficient prices or by not placing orders, so that no efficient price discovery process takes place. In these periods, the efficiency of the opening price is lower than in the nontransparent auction. Regression analysis furthermore shows that this lower efficiency is not corrected in the subsequent continuous double auction. In fact, the higher the MRE value at the opening of the transparent auction compared to the nontransparent auction, the higher is the difference between the MRE values in the continuous double auction.

Further experimental analysis concerning the transparency of the opening call auctions would therefore be valuable. Note that most stock exchanges outside the lab use lower levels of transparency than that chosen in our transparent call auction treatment. It would therefore be worthwhile to learn whether the higher efficiency in our nontransparent call auction markets is a corner solution, or whether there is an optimal level somewhere in between no and full transparency. Such a U-shaped pattern is, for example, suggested by Comerton-Forde and Rydge [41]. A study investigating this question could, for example, only provide traders with indicative prices (this is, e.g., the case at the Deutsche Börse) or show a limited number of bids (this is the case, e.g., at Euronext Paris and at the New York Stock Exchange). Furthermore, manipulative behavior by some traders, as seems to be present in a few periods of our study, could be analyzed by further experiments with low price signals for one part of traders and high price signals for the second part of traders. Therefore, it could be analyzed whether manipulating behavior in a transparent auction can generally be observed. This would be particularly interesting in a setting with heterogeneously informed traders.

As a final area of possible future research we identify the implementation of a market making setting in the continuous trading phase (like, e.g., at NASDAQ, cf. the empirical work of [24]). In some empirical and experimental studies (e.g., [16, 49]), market making without an opening call auction exhibits lower market quality than the continuous double auction. An opening call auction could therefore possibly have an even greater spillover effect in this kind of institution.

## Figures and Tables

**Table 1:** Performance-dependent payout structure of the experiment.

Ranking	Complement markets	CDA alone
1	47.00€	40.00€
2	35.00€	30.00€
3	26.00€	22.00€
4	19.50€	16.50€
5	14.50€	12.00€
6	10.50€	8.50€
7	7.50€	6.10€
8	5.00€	4.10€
9	3.00€	2.50€
10	1.50€	1.30€
11	0.50€	0.50€
12	0.00€	0.00€

**Table 2:** Regression analysis market open.

	Model (1)	Model (2)	Model (3)
CM	−0.020 (0.006)^***^	−0.016 (0.005)^***^	−0.022 (0.004)^***^

CMT	−0.013 (0.007)^**^	−0.010 (0.006)^*^	−0.024 (0.004)^***^

Session dummies, period dummies, and interactions	Yes	Yes	Yes

Const.	0.087 (0.029)^***^	0.058 (0.007)^***^	0.076 (0.009)^***^

*R* ^2^	0.39	0.36	0.56

Adj. *R* ^2^	0.08	0.03	0.34

*N*	126	126	126

^*∗*^ *P* < 0.1; ^*∗∗*^ *P* < 0.05; ^*∗∗∗*^ *P* < 0.01. Standard errors clustered at the session level (in parentheses). Session and period dummy interaction coefficients omitted.

**Table 3:** Descriptive statistics of period and last trade MRE^CDA^ values as well as results of Wilcoxon rank sum tests between the stand-alone CDA and the CDA of the complement markets.

Panel A: descriptive statistics	CDA alone	CDA-CM	CDA-CMT
Mean	3.60%	2.68%	2.82%
Median	3.22%	2.65%	3.15%
Standard deviation	1.95%	1.15%	1.15%
Mean (last trade)	2.93%	2.27%	2.64%
Median (last trade)	2.50%	2.46%	2.65%

Panel B: Wilcoxon tests	CDA alone versus CDA-CM	CDA alone versus CDA-CMT	CDA-CM versus CDA-CMT

*Z*	1.725	1.214	−0.958
*P* value	0.085	0.225	0.338
*Z* (last trade)	1.214	0.319	−0.831
*P* value (last trade)	0.225	0.749	0.401

**Table 4:** Regression analysis spillover effect.

	Model (4)	Model (5)	Model (6)	Model (7)	Model (8)
CM	−0.009 (0.003)^***^	−0.005 (0.003)	−0.017 (0.004)^***^	−0.013 (0.005)^***^	−0.014 (0.004)^***^

CMT	−0.008 (0.003)^**^	−0.005 (0.003)	−0.006 (0.004)	−0.002 (0.005)	−0.004 (0.005)

RE_*o*,*t*_		0.209 (0.057)^***^			0.187 (0.072)^**^

*S*_*t*_^Call^				0.186 (0.120)	

Session dummies, period dummies, and interactions	Yes	Yes	Yes	Yes	Yes

Const.	0.050 (0.021)^**^	0.032 (0.014)^**^	0.066 (0.012)^***^	0.052 (0.014)^***^	0.050 (0.010)^***^

*R* ^2^	0.41	0.52	0.51	0.52	0.54

Adj. *R* ^2^	0.11	0.25	0.25	0.26	0.29

*N*	126	126	126	126	126

^*∗*^ *P* < 0.1; ^*∗∗*^ *P* < 0.05; ^*∗∗∗*^ *P* < 0.01. Standard errors clustered at the session level (in parentheses). Coefficients for session and period dummies and their interactions omitted.

**Table 5:** Average RE^CDA^ values and results of corresponding Wilcoxon rank sum tests. Results without the first CDA session in parentheses.

Panel A: descriptive statistics	CDA alone	CDA-CM	CDA-CMT
(1) Avg. RE^CDA^, Period 1	4.96% (4.29%)	2.59%	2.51%
(2) Avg. RE^CDA^, Period 2	4.95% (4.21%)	3.24%	2.99%
(3) Avg. RE^CDA^, Period 3	3.24% (2.66%)	2.84%	2.70%
(4) Avg. RE^CDA^, Period 4	2.57% (2.83%)	1.98%	3.06%
(5) Avg. RE^CDA^, Period 5	2.84% (2.86%)	2.40%	2.90%
(6) Avg. RE^CDA^, Period 6	3.05% (3.04%)	3.05%	3.12%
(7) Avg. RE^CDA^, last transaction, and all periods	2.93% (2.67%)	2.27%	2.64%
(8) Avg. RE^CDA^, last transaction, and Period 6	2.41% (2.69%)	2.92%	2.39%

Panel B: Wilcoxon tests	CDA alone versus CDA-CM	CDA alone versus CDA-CMT	CDA-CM versus CDA-CMT

*P* value (1)	0.035 (0.063)	0.025 (0.046)	0.848
*P* value (2)	0.048 (0.087)	0.085 (0.153)	0.848
*P* value (3)	0.655 (1.000)	0.848 (0.475)	0.949
*P* value (4)	0.277 (0.153)	0.482 (0.775)	0.110
*P* value (5)	0.338 (0.317)	0.749 (0.886)	0.482
*P* value (6)	0.949 (1.000)	0.949 (0.886)	0.655
*P* value (7)	0.247 (0.386)	0.682 (0.931)	0.420
*P* value (8)	0.338 (0.475)	0.949 (0.886)	0.949

**Table 6:** Descriptive statistics of period spreads and results of Wilcoxon rank sum tests on average series spreads between the stand-alone CDA and the CDA of the complement markets.

Panel A: descriptive statistics	CDA alone	CDA-CM	CDA-CMT
Mean	4.53%	2.78%	3.91%
Median	4.25%	2.62%	3.27%
Standard deviation	2.21%	1.66%	2.43%

Panel B: Wilcoxon test	CDA alone versus CDA-CM	CDA alone versus CDA-CMT	CDA-CM versus CDA-CMT

*Z*	2.108	1.086	−0.831
*P* value	0.035	0.278	0.406
